# Analogue simulation of pharyngeal airflow response to Twin Block treatment in growing patients with Class II^1^ and mandibular retrognathia

**DOI:** 10.1038/srep26012

**Published:** 2016-05-18

**Authors:** Liang Li, Wei Wu, Guijun Yan, Li Liu, Hong Liu, Guojv Li, Jing Li, Dongxu Liu

**Affiliations:** 1 Department of Orthodontics, College of Stomatology, Shandong University, Shandong Provincial Key Laboratory of Oral Tissue Regeneration, Jinan, 250012, China; 2 Department of Orthodontics, Yantai Stomatological Hospital, Yantai, 264000, China; 3 Department of Stomatology, Weifang People’s Hospital, Weifang, 261041, China; 4 Department of Stomatology, the Affiliated Hospital of Qingdao University, Qingdao, 266000, China; 5 College of Stomatology, Shandong University, Jinan, 250012, China

## Abstract

The flow dynamics of respiratory airflow is the basic factor that influences the ventilation function of the upper airway. This research aimed to investigate the pharyngeal flow field characteristics after Twin Block (TB) treatment in growing patients with Class II^1^ and mandibular retrognathia by computation fluid dynamics (CFD) simulation. Cone beam computed tomography (CBCT) scans of patients who have completed TB treatment (n = 30) and about to accept TB treatment (n = 30) were reconstructed. After CFD simulation, correlations between the pharyngeal pressure drop and morphological parameters were further analyzed. During inspiration, we found that the pressure minimum occurred in the hypopharynx, while the maximum pressure drop and velocity was located in the oropharynx. After TB treatment, the oropharynx and hypopharynx showed significant differences in airflow features, and the most obvious change was observed in the oropharynx. A significant correlation was discovered between the change amount of oropharyngeal pressure drop and volume (r = 0.694, p = 0.001), mean cross-sectional area (r = 0.859, p = 0.000), and ratio of the minimum and mean cross-sectional area (r = 0.898, p = 0.000) of the oropharynx. Our research suggested that the pharyngeal airflow characteristics response positively to mandibular advancement with the enlargement in volume, cross-sectional area and more uniform oropharyngeal area distribution.

Angel Class II^1^ malocclusion with mandibular retrognathia is a common dento-facial deformity in Chinese children. It is characterized by skeletal retrusion of mandible with skeletal or dentoalveolar protrusion of maxillary^1^,^2^. Three-dimensional (3D) imaging studies of upper airway have shown that patients with retruded mandible have a significantly narrowed pharyngeal space compared with normal subjects, which was attributed to a more backward position of the hyoid bone^3^,^4^. Some researchers reported that an inadequate airway may influence nasal respiratory function and even induce symptoms of obstructive sleep apnea syndrome (OSAS) in growing patients^5^,^6^. Therefore, early diagnosis and proper functional appliance therapy in growth period are indispensable for correcting this type of malocclusion.

Twin Block (TB) is one of the popular functional appliances attributing to its high patient acceptability and the ability in producing rapid changes. The appliance is mainly applied to the mandibular skeletal retrognathia patients who are in the peak period of growth and development. It was proved to be an effective method in promoting mandibular advancement and harmonizing facial profile, and thereby has been widely used in clinical orthodontics^7^,^8^.

Previous studies investigated the effect of mandibular position on upper airway size in adult patients, and discovered that the upper airway showed a significant expansion after mandibular advancement treatment^9^,^10^. Many studies evaluated the pharyngeal flow dynamics response followed by the morphological changes in adult patients with OSAS^11^,^12^,^13^. They reported that the upper airway resistance is inversely correlated with the pharyngeal dimension. W. Vos. *et al.* measured the anatomical properties and simulated the airway flow of the upper airway in OSAS patients, and confirmed that there exists a relationship between the morphological parameters of the upper airway and apnea hypopnea index (AHI) in these patients^14^. However, there have been no studies related to pharyngeal flow field characteristics due to morphological changes after mandibular advancement in growing patients. Since the upper airway of children is anatomically different from that of the adults^15^, simulation of the upper airway response to mandibular advancement in growing patients with Class II^1^ and mandibular retrognathia needs to be established.

Computational Fluid Dynamics (CFD) is an appropriate method in analogue simulating the flow field characteristics of the upper airway. It could accurately calculate the functional parameters such as pressure drop and flow resistance before and after treatment based on the patient specific geometrical models of the upper airway derived from 3D imaging technology^16^,^17^,^18^. Thereby provide a better understanding in the effect of the morphological and respiratory airflow changes on the pharyngeal function. Therefore, the objective of this study is to evaluate the pharyngeal airflow response to TB treatment in growing patients with class II^1^ and mandibular retrognathia by means of CFD analogue simulation.

## Methods

The study was critically reviewed and approved by the Ethical Committee of Shandong University Dental School. All the written informed consents were received from parents or legal guardians of the patients, and the study was conducted according to the tenets of the Declaration of Helsinki for research involving human subjects. The methods were carried out in accordance with the approved guidelines of scientific reports.

For the subjects involved in our research are growing patients, their personal growth development have a negligible impact on the upper airway during treatment period^19^. For this reason, a control group is needed. Body mass index (BMI) was calculated for each patient to quantify the development status. Thirty growing patients (13 boys and 17 girls, mean age 11.57 ± 0.94 years, BMI 17.86 ± 1.25) who were diagnosed as class II^1^ malocclusion with mandibular retrognathia and have finished TB treatment were randomly selected as TB group. The treatment period of TB group was 13.67 ± 1.51 months. The control group (13 boys and 17 girls, mean age 11.72 ± 0.86 years, BMI 18.14 ± 0.97) included the patients with the same diagnosis and matched well with the patients of TB group through age, gender, BMI and growth pattern. By this method, factors that influence the upper airway anatomy and morphology other than appliance therapy could be excluded. All the patients had no other potential airway abnormalities.

### Image acquisition and 3D model reconstruction

All the CBCT scans were performed using the CBCT scanner (KaVo Dental GmbH, Bismarckring, Germany) on each wake patient with the Frankfort horizontal plane parallel to the floor. During scanning, patients were required to close their mouth with maximum intercuspation and the tongue was in contact with anterior palate. The scanning area was from the basis cranii level to the third cervical vertebra level (scan time: 8.9 seconds; slice thickness: 0.4 mm, 120 kV, 5 mA). The CBCT data were saved as DICOM (Digital Imaging and Communications in Medicine) format. The pre- and post-treatment CBCT scans of TB group were collected as T1 and T2 data, while the scans of control group before function therapy were collected as control data.

All 3D models of the upper airway in TB and control groups were reconstructed using MIMICS 16.0 (Materialism’s Interactive Medical Image Control System) software. The upper airway was highlighted by setting the threshold between −1024 Hounsfield Units (HU) and −480 HU. The paranasal sinuses were erased manually for there were almost no airflow in the cavity. After image segmenting and region growing, the patient-specific upper airway 3D geometry was obtained.

**Figure 1:**
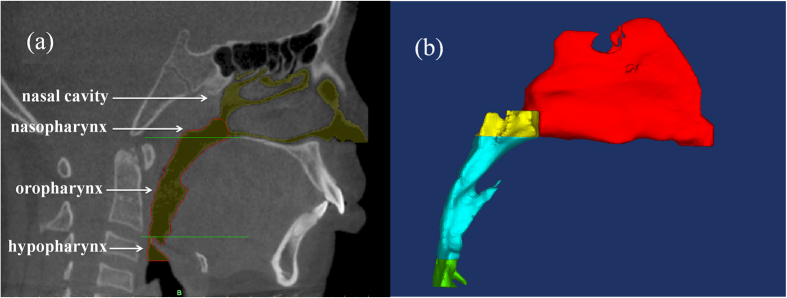
(**a**) The upper airway was divided into nasal cavity, nasoparynx, oropharynx, and hypopharynx and (**b**) 3D model of each section was reconstructed respectively.

The upper airway model consists of nasal cavity and pharynx. After identifying the basis cranii, the PNS point (posterior nasal spine), the superior border of the epiglottis and the C_3_ point (the most anterior inferior point of the third cervical vertebra) in the midsagittal plane, the pharynx is further divided into three parts: the nasopharynx, oropharynx, and hypopharynx by the corresponding cross-sectional slices (Fig. 1). All 3D models were exported as stereolithography (STL) files.

### CFD mesh generation

**Figure 2:**
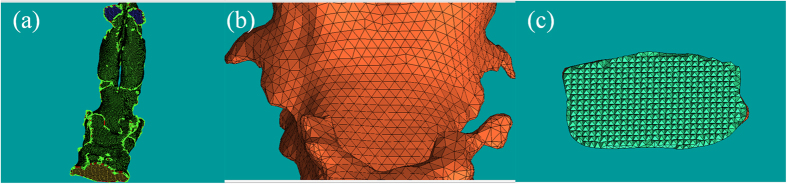
Mesh generation of the upper airway 3D geometry in (**a**) overall, (**b**) magnified, and (**c**) cross-sectional view.

After 3D reconstruction, the upper airway geometry of STL format was loaded into ANSYS ICEM CFD (ANSYS 12.0) for model trimming and mesh generation. The cross-sections of nostrils were selected as the inlet of the upper airway, while the outlet was located at the cross-section in C_3_ level. The whole pharyngeal cavity was defined as body of the geometry. Then, an unstructured tetrahedral volume mesh was generated in the 3D upper airway model to form a computational domain of fluid field (Fig. 2). The maximum grid edge length was set as 1.0 mm while the minimum was 0.5 mm, which resulted in a computational grid of pre-, post-treatment and the control models typically consisting of 8.81 ± 0.09 billion, 12.04 ± 0.10 billion and 9.63 ± 0.08 billion computational cells, respectively. The maximum scale factor of the grids was 0.8.

### Airflow simulation

The 3D mesh was exported and read into a Reynolds Average Navier Stokes CFD solver (Fluent 14.0, Fluent Inc.) for numerical simulation. The air was assumed to be a newtonian and homogeneous fluid. Considering the Mach number of airflow during eupnea is smaller than 0.3, the flow field within the upper airway was incompressible. Pressure boundary conditions are applied for the upper airway constriction have no impact on the static pressure difference between the lungs and atmosphere. A standard atmospheric pressure (gauge pressure was 0 Pa) was set for the inlet and in the outlet, a pressure of −20 Pa was applied. The wall surface of the geometry was assumed to be stationary and non-slip. The effects of the temperature, humidity and vibrissae factors on the fluid were neglected in the simulation, while the gravity factor was included.

A maximum inlet volume flow rate 166 ml/s (10 L/min) was set for the inhaled airflow in an awakened state^18^. The maximum and minimum values of pressure and velocity were artificial limited to unified the reference legends. Laminar approach was selected to model the pharyngeal airflow. The calculation residual was set as 10^−3^ and the iteration numbers were 1000 steps. Second-order discretization schemes were used and the pressure-velocity coupling was solved through the SIMPLE scheme.

### Outcome parameters

The upper airway morphology was quantified by volume (V) and mean cross-sectional area (S_mean_) of each section. The minimum cross-sectional area (S_min_) could be defined and S_min_/S_mean_ was calculated to determine the pharyngeal morphological uniform^11^. The pharyngeal shape was determined by ratio of the largest lateral (LR) and anteroposterior (AP) dimensions (LR/AP) of each cross-sectional slice.

After simulation of the airflow, all the cross sections from nasopharynx to hypopharynx were selected along Z-axis every 1 mm for parameters calculation. The maximum pressure (P_max_), minimum pressure (P_min_) and maximum velocity (v_max_) of the upper airway were extracted from the outcome of the CFD analysis. The airflow pressure drop (ΔP) is computed by ΔP = P_max_ − P_min_, while the pharyngeal resistance (R) is obtained through the formula: R = Q/ΔP (Q was pharyngeal inlet volume flow rate). As Q was constant in our study, ΔP could directly represent the change of R in the following data statistics. All the parameters were measured three times by the same researcher, and the average value was applied to the study.

### Statistical analysis

All the measurements were repeated for each randomly selected patient, with a 1-week interval, to assess intra-rater reliability through paired t-test. The method error (ME) was calculated as: 
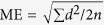
 (d is deviation between the two measurements; n for the number of paired double measurements)^20^.

The final measuring values were loaded into SPSS 17.0 for statistical analysis. The mean value and standard deviation (SD) were calculated for each variable. The statistical differences between the flow dynamics parameters of the upper airway in TB group were analyzed by paired-t test, while the T2 and control data were compared by the independent t-test. By this way, sections with the most significant changes in flow field characteristics of the upper airway due to Twin Block treatment could be found out. The relationship between the pharyngeal pressure drop and morphological parameters (V, S_mean_, S_min_/S_mean_, LP/AP) were further assessed by Pearson correlation coefficient.

## Results

The age distribution and BMI showed no statistical differences between the TB and control group (p = 0.914, 0.852). There were no significant differences between the first and repeated measurements (p = 0.873), which proved high intra-rater reliability. The maximum ME of pharyngeal volume and area measurements were 0.21 mm^3^ and 7.85 mm^2^, respectively. In the numerical simulation of pharyngeal airflow, the maximum ME of pressure measurements was 1.65 Pa, while for velocity measurements was 0.59 m/s.

Table 1 gives an overview of the measured and computed hydrodynamic parameters in the numerical simulation. The inspiratory pressure falls continuously with the respiratory airflow move downwards from the nasal cavity in all subjects. The pressure minimum occurs in the hypopharynx while airflow travels around the outlet of the pharyngeal model. The maximum pressure drop and velocity, however, were located in the oropharynx region where the minimum cross-section area lies in all of the subjects modeled.

The airflow characteristics of the oropharynx and hypopharynx changed obviously through comparing the T2 data of TB group and the control data, while the nasopharyngeal flow field shows no statistical differences after mandibular advanced treatment. The most significant change of airflow patterns was still observed in the oropharynx.

**Figure 3:**
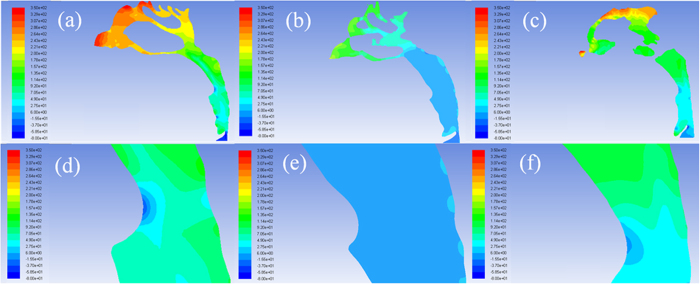
The pressure profile of the pharyngeal airflow in (**a**) pre- and (**b**) post-treatment of TB group and (**c**) control group, and magnified view of the narrow region in (**d**) pre- and (**e**) post-treatment of TB group and (**f**) control group.

**Figure 4:**
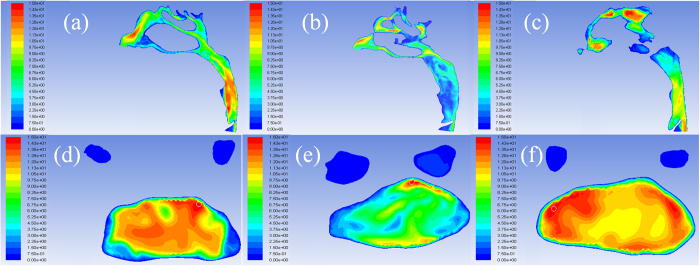
The velocity profile of the pharyngeal airflow in (**a**) pre- and (**b**) post-treatment of TB group and (**c**) control group, and magnified view of the minimum cross-section in (**d**) pre- and (**e**) post-treatment of TB group and (**f**) control group.

Figure 3a–c shows the changes in pressure profile of the airflow with the variation of the pharyngeal morphology. The pressure gradient in the narrowest region of oropharynx was significantly lower in the T2 data as the oropharyngeal pressure drop decreased from 67.43 ± 15.12 Pa to 40.18 ± 8.26 Pa after TB treatment (Fig. 3d–f). From Fig. 4a significantly reduced number of local eddy was also observed in the oropharynx of T2 data as the velocity of airflow slipped from 11.24 ± 1.92 m/s to 7.14 ± 1.92 m/s after mandibular repositioning. In addition, we noticed that as the inhaled airflow accelerates through the oropharynx and into the narrowest region, the most negative pressure and the maximum velocity were generally located along the anterior wall of the upper airway. A significant negative correlation was observed in the change amount of oropharyngeal pressure drop and V, S_mean_, and S_min_/S_mean_ of the oropharynx (Table 2).

## Discussion

In this study, we explored the biomechanical reaction of obstructed upper airway using patient-specific 3D CFD models derived from CBCT scans before and after mandibular advancement. It is shown that the flow field inside the pharynx especially in oropharynx response positively to TB treatment in growing patients with class II^1^ and mandibular retrognathia. In addition, a significant correlation was observed between the upper airway patency and V, S_mean_ and S_min_/S_mean_ of the oropharynx, suggesting that the anatomic morphology may predict the functional properties of the upper airway in clinical treatment.

During inspiration, as increase of the kinetic energy and dissipative energy loss in the respiratory airflow upstream, the pharyngeal luminal pressure decreases generally from 0 Pa in nostrils and reaches subatmospheric values in the oropharynx and hypopharynx region^21^. The pressure gradient was relatively large in the oropharynx for the maximum pressure drop that reflects airway resistance and quantitatively indicates the OSAS severity was observed here^18^. whereas, the pressure fluctuation in the narrow region of oropharynx was negligible and the pressure drop became much lower after mandibular advancement compared to the pre-treatment and the control group. This may result in decrease in pharyngeal compliant and thereby an improvement in pharyngeal collapse prevention capability^22^. The expansion in the oropharyngeal space could help reduce the kinetic energy of airflow and maintain a higher luminal pressure that permit a freer passage of air^21^.

Given the inhaled volume flow rate, the respiratory airflow velocity reached a peak value in the narrow region of the oropharynx. This is consistent with previous studies about adult OSAS patients, showing that the maximum velocity of airflow occurred in the velopharynx, the most occlusive area of the upper airway^23^,^24^. The restricted region of the oropharynx could directly induce a jet flow with significant increase in the pressure drop as well as the airflow velocity, as a linear correlation has been proved between the flow rate and the square root of the maximum pressure drop in several flow sensitive studies^18^,^25^.

The wall shear stresses, which depended on the pharyngeal airflow, was the crucial factor controlling the defense mechanisms of the upper airway^26^,^27^. For patients with narrowed airway, the excessive wall shear stresses generated by the high speed airflow along the pharyngeal wall may adversely affect the functioning of epithelial cells^28^. Children with insufficient neuromuscular response that maintains airway patency may result in airway collapse and even induce OSAS^21^,^29^. In our study, the airflow became more smoothly after TB treatment may indicate a significantly lower level of wall shear stresses that enhance epithelial barrier function and thus draw a protection on the mucous membrane of the pharyngeal wall^27^,^30^.

V, S_mean_ and S_min_/S_mean_ are discovered as good parameters to predict the changes in the upper airway resistance, which confirms the results of previous researches^11^,^31^,^32^,^33^. Holsbeke. *et al.*^11^ pointed out that S_min_ and S_min_/S_mean_ play a more dominant role in the decrease of pharyngeal resistance than other morphological parameters, as S_min_ is more sensitive to the rotation of the tongue that caused by mandibular repositioning. Whereas, an individual difference in the location and size of S_min_ in eupnoea was reported in the research of Ye. *et al.*^34^. They reckoned that the measurement errors between individuals in different measuring planes could be effectively reduced by calculation of S_min_/S_mean_. In the present study, S_min_/S_mean_ shows a strong link with pharyngeal patency, indicating that the upper airway with a rather uniform area distribution react more positively than pre-treated airways which are more concave and obstructed.

The nasopharynx was the only segment that showed no statistical differences in flow field features after CFD simulation. A relatively constant anatomical morphology in nasopharynx due to TB treatment was reported in our published research^35^, which further confirmed the interaction between the pharyngeal shape and ventilation function of the upper airway.

In our research, the numerical simulation was conducted with several limiting but reasonable assumptions, including laminar model, steady-state airflow and neglecting the influence of temperature and humidity. In the particle imaging velocimetry (PIV) experiment of Hörschler. *et al.*^36^, the respiratory airflow state was proved to be laminar in quiet inspiratory and expiratory. The steady-state airflow inside the upper airway was verified through steady and unsteady computation in a comparative research between growing OSAS patients and normal children^33^. The temperature and humidity of airflow were considered as negligible factors for no significant effects were discovered on the pharyngeal flow field in a scaled up model of nasopharynx^37^. In spite of the limitations, these assumptions make the complicated flow geometry tractable since the flow field inside the pharynx is time varying and unknown and the flow rate measurements were not available for the patients involved.

## Conclusion

The pharyngeal airflow pressure distribution and resistance response positively after mandibular advancement with the enlargement in volume, cross-sectional area and more uniform area distribution, and ultimately result in an improvement in ventilation function of the upper airway in growing patients with class II^1^ and mandibular retrognathia.

## Additional Information

**How to cite this article**: Li, L. *et al.* Analogue simulation of pharyngeal airflow response to Twin Block treatment in growing patients with Class II^1^ and mandibular retrognathia. *Sci. Rep.* **6**, 26012; doi: 10.1038/srep26012 (2016).

## Figures and Tables

**Table 1:** The pharyngeal hydrodynamic parameters of TB and control group.

Variables		TB group		Control group	p value	
		T1	T2		T1 & T2	T2 & Control
Na-P_min_ (Pa)	mean	16.36	14.72	12.66	0.191	0.226
	SD	5.29	4.84	4.20		
Na-v_max_ (m/s)	mean	6.25	6.83	6.96	0.496	0.615
	SD	2.93	3.07	2.78		
Na-ΔP (Pa)	mean	38.14	35.93	36.72	0.332	0.269
	SD	9.56	8.26	9.19		
Or-P_min_ (Pa)	mean	−30.97	−12.66	−20.73	0.005	0.019
	SD	7.46	4.89	6.53		
Or-v_max_ (m/s)	mean	11.24	7.14	8.69	0.012	0.037
	SD	3.36	1.92	2.16		
Or-ΔP (Pa)	mean	67.43	40.18	53.25	0.004	0.020
	SD	15.12	8.26	10.73		
Hy-P_min_ (Pa)	mean	−48.90	−43.96	−46.46	0.524	0.416
	SD	10.02	9.76	9.93		
Hy-v_max_ (m/s)	mean	8.07	6.46	7.54	0.039	0.042
	SD	3.14	2.75	3.02		
Hy-ΔP (Pa)	mean	45.36	37.45	41.79	0.025	0.036
	SD	10.01	9.36	9.60		

(Na, nasopharyngeal; Or, oropharyngeal; Hy, hypopharynxgeal).

**Table 2:** Correlations between oropharyngeal pressure drop and morphological parameters.

Variables		Or-V (T1-T2)	Or-S_mean_ (T1-T2)	Or-LR/AP (T1-T2)	Or-S_min_/S_mean_ (T1-T2)
Or-ΔP (T1-T2)	*r*	0.694	0.859	0.117	0.898
	*p*	0.001	0.000	0.537	0.000

## References

[b1] Pancherz H., Zieber K. & Hoyer B. Cephalometric characteristics of Class II division 1 and Class II division 2 malocclusions: a comparative study in children. Angle Orthod. 67, 111–120 (1997). 9107375 10.1043/0003-3219(1997)067<0111:CCOCID>2.3.CO;2

[b2] Hitchcock H. P. A cephalometric description of Class II, Division 1 malocclusion. Am. J. Orthod. 63, 414–423 (1973). 4511353 10.1016/0002-9416(73)90146-2

[b3] Abdelkarim A. A cone beam CT evaluation of oropharyngeal airway space and its relationship to mandibular position and dentocraniofacial morphology. JWFO 1, 55–59 (2012).

[b4] Hakan E. L. & Palomo J. M. Airway volume for different dentofacial skeletal patterns. Am. J. Orthod. Dentofacial Orthop. 139, 511–521 (2011). 10.1016/j.ajodo.2011.02.015 21640863

[b5] Vieira B. B. *et al.* Cephalometric evaluation of facial pattern and hyoid bone position in children with obstructive sleep apnea syndrome. Int. J. Pediatr. Otorhinolaryngol. 75, 383–386 (2011). 21216478 10.1016/j.ijporl.2010.12.010

[b6] Hong J. S., Oh K. M., Kim B. R., Kim Y. J. & Park Y. H. Three-dimensional analysis of pharyngeal airway volume in adults with anterior position of the mandible. Am. J. Orthod. Dentofacial Orthop. 140, 161–169 (2011). 10.1016/j.ajodo.2011.04.020 21967954

[b7] Surana A., Chakrabarty S. & Dhar S. Correction of skeletal Class II Malocclusion using Functional-Fixed Appliance Therapy. J. Ind. Orthod. Soc. 46, 348–351 (2012).

[b8] Thiruvenkatachari B., Sandler J., Murray A., Walsh T. & O’Brien K. Comparison of Twin-block and Dynamax appliances for the treatment of Class II malocclusion in adolescents: A randomized controlled trial. Am. J. Orthod. Dentofacial Orthop. 138, 144.e1-9 (2010). 10.1016/j.ajodo.2010.01.025 20691354

[b9] Ogutcen-Toller M., Sarac Y. S., Cakır-Ozkan N., Sarac D. & Sakan B. Computerized tomographic evaluation of effects of mandibular anterior repositioning on the upper airway: A pilot study. J. Prosthet. Dent. 92, 184–189 (2004). 15295329 10.1016/j.prosdent.2004.05.003

[b10] Haskell J. A. *et al.* Effects of Mandibular Advancement Device (MAD) on Airway Dimensions Assessed With Cone-Beam Computed Tomography. Semin. Orthod. 15, 132–158 (2009).

[b11] Holsbeke C. V. *et al.* Anatomical and functional changes in the upper airways of sleep apnea patients due to mandibular repositioning: A large scale study. J. Biomech. 44, 442–449 (2011). 20970801 10.1016/j.jbiomech.2010.09.026

[b12] Zhao M., Barber T., Cistulli P. A., Sutherland K. & Rosengarten G. Simulation of upper airway occlusion without and with mandibular advancement in obstructive sleep apnea using fluid-structure interaction. J. Biomech. 46, 2586–2592 (2013). 24035015 10.1016/j.jbiomech.2013.08.010

[b13] De Backer J. W. *et al.* Functional imaging using computational fluid dynamics to predict treatment success of mandibular advancement devices in sleep-disordered breathing. J. Biomech. 40, 3708–3714 (2007). 17663990 10.1016/j.jbiomech.2007.06.022

[b14] Vos W. *et al.* Correlation between severity of sleep apnea and upper airway morphology based on advanced anatomical and functional imaging. J. Biomech. 40, 2207–2213 (2007). 17178125 10.1016/j.jbiomech.2006.10.024

[b15] Goldstein N. A. & Tomaski S. M. Embryology and anatomy of the mouth, pharynx and esophagus. Pediatric otolaryngology 2, 1083–1102 (2003).

[b16] Mylavarapu G. *et al.* Validation of computational fluid dynamics methodology used for human upper airway flow simulations. J. Biomech. 42, 1553–1559 (2009). 19501360 10.1016/j.jbiomech.2009.03.035

[b17] Yu C. C. *et al.* Computational fluid dynamic study on obstructive sleep apnea syndrome treated with maxillomandibular advancement. J. Craniofac. Surg. 20, 426–430 (2009). 19305244 10.1097/SCS.0b013e31819b9671

[b18] Zhao M., Barber T., Cistulli P., Sutherland K. & Rosengarten G. Computational fluid dynamics for the assessment of upper airway response to oral appliance treatment in obstructive sleep apnea. J. Biomech. 46, 142–150 (2013). 23218140 10.1016/j.jbiomech.2012.10.033

[b19] Li H., Lu X., Shi J. & Shi H. Measurements of normal upper airway assessed by 3-dimensional computed tomography in Chinese children and adolescents. Int. J. Pediatr. Otophi. 75, 1240–1246 (2011). 10.1016/j.ijporl.2011.06.022 21816490

[b20] Houston W. J. B. The analysis of errors in orthodontic measurements. Am. J. Orthod. 83, 382–390 (1983). 6573846 10.1016/0002-9416(83)90322-6

[b21] Xua C. *et al.* Computational fluid dynamics modeling of the upper airway of children with obstructive sleep apnea syndrome in steady flow. J. Biomech. 39, 2043–2054 (2006). 16098533 10.1016/j.jbiomech.2005.06.021

[b22] Lu L. Y. *et al.* Establishment and application of CFD method in OSAHS patients. J. Oral Maxil. Surg. 10, 397–402 (2012).

[b23] Sung S. J., Jeong S. J., Yu Y. S., Hwang C. J. & Pae E. K. Customized three-dimensional computational fluid dynamics simulation of the upper airway of obstructive sleep apnea. Angle Orthod. 76, 791–799 (2006). 17029512 10.1043/0003-3219(2006)076[0791:CTCFDS]2.0.CO;2

[b24] Jeong S. J., Kim W. S. & Sung S. J. Numerical investigation on the flow characteristics and aerodynamic force of the upper airway of patient with obstructive sleep apnea using computational fluid dynamics. Med. Eng. Phy. 29, 637–651 (2007). 10.1016/j.medengphy.2006.08.017 17049904

[b25] Xu C. *et al.* Computational fluid dynamics modeling of the upper airway of children with obstructive sleep apnea syndrome in steady flow. J. Biomech. 39, 2043–2054 (2006). 16098533 10.1016/j.jbiomech.2005.06.021

[b26] Sul B., Wallqvist A., Morris M. J., Reifman J. & Rakesh V. A computational study of the respiratory airflow characteristics in normal and obstructed human airways. Computers in Biology and Medicine 52, 130–143 (2014). 25058489 10.1016/j.compbiomed.2014.06.008

[b27] Sidhaye V. K., Schweitzer K. S., Caterina M. J., Shimoda L. & King L. S. Shear stress regulates aquaporin-5 and airway epithelial barrier function. Proc. Natl. Acad. Sci. 105, 3345–3350 (2008). 18305162 10.1073/pnas.0712287105 PMC2265191

[b28] Huh D. *et al.* A coustically detectable cellular-level lung injury induced by fluid mechanical stresses in microfluidic airway systems. Proc. Natl. Acad. Sci. 104, 18886–18891 (2007). 18006663 10.1073/pnas.0610868104 PMC2141877

[b29] Marcus C. L. *et al.* Upper airway dynamic responses in children with the obstructive sleep apnea syndrome. Pediatric Research 57, 99–107 (2004). 15557113 10.1203/01.PDR.0000147565.74947.14

[b30] Li Y., Li Y., Chen J., Huo Z. & Li B. The change of morphology and airflow dynamics in upper airway by the use of oral appliance in OSAHS patients. J. Pract. Stomatol. 30, 183–187 (2014).

[b31] De Backer J. W., Vos W. G., Verhulst S. L. & Backer W. D. Novel imaging techniques using computer methods for the evaluation of the upper airway in patients with sleep-disordered breathing: a comprehensive review. Sleep Med. Rev. 12, 437–447 (2008). 18926741 10.1016/j.smrv.2008.07.009

[b32] Vos W. *et al.* Correlation between severity of sleep apnea and upper airway morphology based on advanced anatomical and functional imaging. J. Biomech. 40, 2207–2213 (2007). 17178125 10.1016/j.jbiomech.2006.10.024

[b33] Xu C. *et al.* Computational fluid dynamics modeling of the upper airway of children with obstructive sleep apnea syndrome in steady flow. J. Biomech. 39, 2043–2054 (2006). 16098533 10.1016/j.jbiomech.2005.06.021

[b34] Ye J., Han D., Wang J., Wang X. & Wang J. Study on topodiagnosis of obstructive sleep apnea syndrome. Chin. Arch. Otolaryngol. Head Neck Surg. 12, 519–522 (2005).

[b35] Li L. *et al.* CBCT Evaluation of the Upper Airway Morphological Changes in Growing Patients of Class II Division 1 Malocclusion with Mandibular Retrusion Using Twin Block Appliance: A Comparative Research. Plos One 8, e94378 (2014). 24705466 10.1371/journal.pone.0094378 PMC3976395

[b36] Hörschler I., Meinke M., Schröder W. & Meinke M. Investigation of the impact of thegeometry on the nose flow. Eur. J. Mechanics B/Fluids 25, 471–490 (2006).

[b37] Haln I., Scherer P. W. & Mozell M. M. Velocity profiles measured for airflow through a large-scale model of the human nasal cavity. J. Appl. Physiol. 75, 2273–2287 (1993). 8307887 10.1152/jappl.1993.75.5.2273

